# Genomic DNA *k*-mer spectra: models and modalities

**DOI:** 10.1186/gb-2009-10-10-r108

**Published:** 2009-10-08

**Authors:** Benny Chor, David Horn, Nick Goldman, Yaron Levy, Tim Massingham

**Affiliations:** 1 School of Computer Science, Tel Aviv University, Klausner St, Ramat-Aviv, Tel-Aviv 39040, Israel; 2 School of Physics and Astronomy, Tel Aviv University, Klausner St, Ramat-Aviv, Tel-Aviv 39040, Israel; 3 European Bioinformatics Institute, Hinxton, Cambridge, CB10 1SD, UK

## Abstract

**Background:**

The empirical frequencies of DNA *k*-mers in whole genome sequences provide an interesting perspective on genomic complexity, and the availability of large segments of genomic sequence from many organisms means that analysis of *k*-mers with non-trivial lengths is now possible.

**Results:**

We have studied the *k*-mer spectra of more than 100 species from Archea, Bacteria, and Eukaryota, particularly looking at the modalities of the distributions. As expected, most species have a unimodal *k*-mer spectrum. However, a few species, including all mammals, have multimodal spectra. These species coincide with the tetrapods. Genomic sequences are clearly very complex, and cannot be fully explained by any simple probabilistic model. Yet we sought such an explanation for the observed modalities, and discovered that low-order Markov models capture this property (and some others) fairly well.

**Conclusions:**

Multimodal spectra are characterized by specific ranges of values of C+G content and of CpG dinucleotide suppression, a range that encompasses all tetrapods analyzed. Other genomes, like that of the protozoa *Entamoeba histolytica*, which also exhibits CpG suppression, do not have multimodal *k*-mer spectra. Groupings of functional elements of the human genome also have a clear modality, and exhibit either a unimodal or multimodal behaviour, depending on the two above mentioned values.

## Abstract 2

Tetrapods, unlike other organisms, have multimodal spectra of k-mers in their genomes

## Background

The distribution of DNA *k*-mers (DNA 'words' of length *k*) - namely, the *k*-mer spectrum - in whole genome sequences provides an interesting perspective on the complexity of the corresponding species. A number of theoretical investigations of genomic *k*-mer distributions were done prior to the sequencing of large genomes and these works suggested various plausible probabilistic models and parameters for such *k*-mer distributions. Despite the relative abundance of sequenced genomes to date, the number of works investigating empirical *k*-mer distributions for values of *k *exceeding 2 or 3 is not very large. The main emphasis has been on studying words with extreme frequencies, namely, either missing or rare *k*-mers, or those with very high frequencies.

Several models for the distribution of *k*-mers in sequences have been proposed. For example, Robin and Schbath [1] compared several approximate *k*-mer distributions, and also analyzed the empirical *k*-mer distributions of the phage Lambda (48,500 bp genome). Reinert *et al*. [2] discussed various plausible *k*-mer distributions, showing that the distribution for the number of occurrences of a particular *k*-mer in sequences generated from a hidden Markov model has two distinct large-sample regimes: a normal distribution for abundant *k*-mers, and a Poisson or compound Poisson distribution for extremely rare *k*-mers.

With the sequencing of more complete genomes, it has become possible to move from theoretical to empirical studies and to examine the properties of these DNA words and how their distributions vary between different species or genome elements. The most basic empirical question that has been investigated is that of missing DNA *k*-mers. Earlier works have studied non-existent short amino acid *k*-mers [3,4], and have attributed them mainly to chemical constraints (such as hydrophobic and hydrophilic amino acids). DNA does not have the complex three-dimensional structure and chemical constraints of proteins, although the nucleotide composition has been reported by el antri *et al*. [5] to weakly affect the structure of double-stranded DNA. Intuitively, if *k *is not too large compared to the genome or chromosome length, we expect that all *k*-mers will be present. This expectation turns out to be incorrect. Fofanov *et al. *[6] studied correlations between present and absent short DNA *k*-mers in over 1,500 species, and found short *k*-mers that are missing. A systematic study of missing *k*-mers, termed 'nullomers', was carried out by Hampikian and Andersen [7]. They reported the complete lists of missing *k*-mers (8 ≤ *k *≤ 13) in 12 species, including human. Several possible uses of these nullomers were suggested, and it was claimed that 'these absent sequences define the maximum set of potentially lethal oligomers, ..., and identify potential targets for therapeutic intervention and suicide markers.' Herold, Kurtz, and Giegerich [8] developed an efficient algorithm for finding all missing words. Zhou, Olman and Xu [9] studied genome barcodes using fragment size *M *(1,000 ≤ *M *≤ 10,000) and based on *k*-mers (1 ≤ *k *≤ 6). They report that sequences generated by Markov models of order three are the closest to the barcodes of genomic sequences in terms of their appearance. Mrázek and Karlin [10] studied different aspects of large viral genomes. They showed that the low order Markov models identify the most frequent *k*-mers fairly well. In an immunological context, Stacey *et al*. [11] studied CpG suppression in 8-mers of mammalian genomes (mouse, human) and bacterial genomes (*Escherichia coli*). A consequence of their work is that the mammalian spectra are multimodal, while the bacterial spectra are unimodal. In a recent study, Csürös, Noé, and Kucherov [12] explored the empirical *k*-mer genomic spectrum, and suggested a rethinking of the significance of word frequencies. Their study focuses on overabundant *k*-mers, showing that their distribution has a heavy sub-exponential tail. They argue that the frequencies of genomic *k*-mers across all genomes can be described using a double Pareto log-normal (DPLN) distribution [13]; this distribution therefore represents a universal genomic feature. In particular, they claim that DPLN provides a much better fit to the empirical *k*-mer distributions than Bernoulli or first-order Markov models, due to its heavier tail (*k*-mers with extreme abundance are more common). In addition, Csürös *et al*. suggest a simple model of evolution by random duplications ('copy and insert') that they report produced distributions whose tails are similar, in simulations, to the DPLN.

## Results and discussion

### Empirical distributions

**Figure 1:**
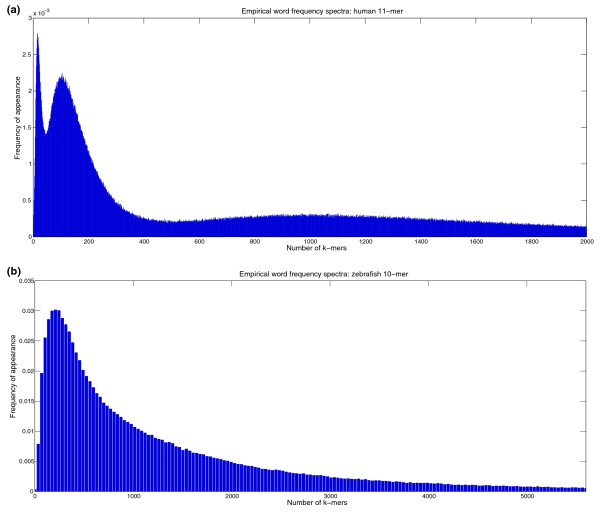
Empirical spectra of human and zebrafish. Empirical word frequency spectra showing the two types of behavior described in this paper. The *x*-axis is the abundance of *k*-mers, and the *y*-axis describes the frequency of words with that abundance in the relevant genome. **(a) **Human genome 11-mers (0 ≤ *x *≤ 2,000 occurrences), exhibiting multimodal behavior. **(b) **Zebrafish genome 10-mer distribution (0 ≤ *x *≤ 5,500 occurrences), with a unimodal distribution.

In this work we study the whole landscape of genomic *k*-mers (4 ≤ *k *≤ 13) across more than 100 species from Archea, Bacteria, and Eukaryota (Additional data file 7). We are interested not only in the high and low ends of the distribution, but in the whole curve, and in particular in the modality of the *k*-mer distributions: does the histogram have a single maximum (unimodal) or multiple local maxima (multimodal)? As can be expected, most species exhibit unimodal *k*-mer distributions. However, a few species, including all mammals, have multimodal *k*-mer spectra, implying that there are distinct groups of common and extremely rare *k*-mers rather than a continually varying distribution. Figure 1 shows two empirical *k*-mer spectra (histograms of *k*-mer abundance) for human genome 11-mers (Figure 1a) and zebrafish genome 10-mers (Figure 1b). The human distribution has three distinct modes, with the maximum of these attained at *x *= 16, *y *= 0.00279 (meaning 0.00279 of all 4^11 ^words, that is, 11,718 words, appear exactly 16 times in the human genome), and the two other local maxima at larger values of *x*.

#### Mammalian genomes

The empirical distributions of *k*-mers in mammalian single chromosomes and whole genomes that we examined were all multimodal. Additional data file 1 depicts the multimodal distributions for human chromosomes 1 (a long chromosome), 6 (medium) and 20 (short), for both 9-mers and 11-mers. Additional data file 2 depicts these distributions for the complete human and opossum genomes, again for both 9-mers and 11-mers. Like the spectrum for 11-mers in the human genome (Figure 1a), there is typically a high peak close to zero, corresponding to a large number of *k*-mers that are either missing or very rare (a low number of appearances), with a second, shallower local peak approximately around the average number of occurrences, from where the numbers decrease monotonically. Often there is a third peak between these two. The high peak close to zero flattens when the length of the genome grows larger compared to 4^*k*^, the number of possible *k*-mers, and conversely gains more mass as *k *increases. The decay of the tail (over-abundant *k*-mers) is slower than exponential, as discussed by Csürös *et al*. [12].

#### Non-mammalian genomes

We analyzed the *k*-mer distributions for 89 non-mammalian genomes: 33 from Archea, 36 from Bacteria, and 20 from non-mammalian Eukaryota, including 8 vertebrates. These distributions can be divided into two main categories, unimodal and multimodal distributions.

##### Unimodal distributions

For unimodal distributions, illustrated for zebrafish in Figure 1b, the corresponding *k*-mer distributions have a single maximum, usually at a relatively low number of *k*-mers. Additional data file 3 depicts typical unimodal *k*-mer distributions for nine species. To account for different genome lengths, we used values of *k *that are ⌈0.7 log_4 _ℓ⌉ or above, where ℓ is the genome length (see the Materials and methods section).

##### Multimodal distributions

For multimodal distributions, the corresponding *k*-mer distributions have two or more maxima. We found that only a small and well characterized group of species exhibits this distribution (Additional data file 4). This group includes chicken (*Gallus gallus*), green anole lizard (*Anolis carolinesis*), and tree frog (*Xenopus tropicalis*), all in the tetrapod clade. Notice that the five bony fish, which are vertebrates but not tetrapods, are not part of this group. Usually one (or more) high maximum exists at a very low number of *k*-mers, and another, shallower one at a larger number.

#### Human genomic regions

The word distributions in specific functional categories of the human genome: exons, introns, 3' untranslated regions (UTRs), 5' UTRs, and gene promoter regions do not all share the same modality as the human chromosomal averages. The gene promoter regions were separately analyzed three times, corresponding to varying lengths of the promoter region (600, 1,000, and 5,000 nucleotide bases upstream of the 5' UTR of the gene). The most striking empirical observation is that the exons, the 5' UTRs, and the shorter lengths (600 and 1,000 bases) of gene promoter regions exhibit unimodal *k*-mer distributions, while the introns, 3' UTRs and the gene promoter regions of length 5,000 bases exhibit multimodal *k*-mer distributions (Additional data file 6).

#### Additional genomes

In addition to the detailed analysis performed on the mammalian and non-mammalian genomes mentioned above, analysis was also performed on 910 bacterial and archeal genomes (all complete microbial genomes listed at [14] and available in the EMBL nucleotide database on 10 August 2009). We created a website [15] that contains the accession number, species name, effective genome length, value 0.7·log_4_(genome length), C+G content, *ρ*_CpG _for that genome, the empirical frequencies of *k*-mers for various values of *k*, parameters for low order Markov models, and spectra plots for various values of *k*. In addition, the site contains the *k*-mer spectra for additional eukaryotic species, including tetrapods (mostly mammals), individual human chromosomes, and different human genomic regions.

### Low-order Markov models

The unexpected finding of different modalities motivated us to ask what probabilistic models could describe these genomic distributions. Perhaps the simplest probabilistic models to describe strings like genomes and chromosomes are low-order Markov models [16], such as those commonly used for a genomic 'background' comparison when searching for regulatory elements (for example, [17,18]). A zero-order Markov model simply describes the frequencies of each nucleotide (when we consider both strands of genomic DNA, the frequencies of A, T are equal, and so are those of C, G) and the underlying model is a Bernoulli sequence. A first-order Markov model describes the frequencies of individual nucleotides given the nucleotide immediately preceding it, a second-order Markov model describes the frequencies of individual nucleotides given the pair of nucleotides immediately preceding it, and so on.

**Figure 2:**
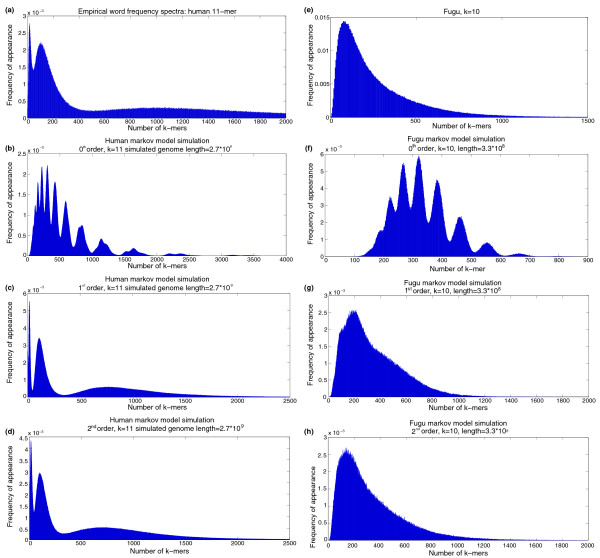
Simulated spectra from Markov models. Markov model simulations of the *k*-mer spectra of **(a-d) **human 11-mers and **(e-h) **Fugu 10-mers. For each species four graphs are shown: the empirical histograms, zero-order Markov model, first-order Markov model, and second-order Markov model. Simulation sequence length was equal to that of the original genome for each species.

The expected occurrence of each *k*-mer from a Markov model can be calculated by noticing that a genomic sequence can be visualized as a random walk around *k*-mer space and the low-order Markov chain is embedded within this larger model. Alternatively, a sequence of the same length as the original genome can be simulated, and its empirical spectrum determined for comparison with the original genome. Zero-order Markov models generate strings that are a poor match to the original genomes, whereas first-order and second-order models do surprisingly well, qualitatively describing the modalities observed in the empirical *k*-mer spectra (Figure 2a-h). To our knowledge it has not been previously appreciated that simple Markov models can generate complex multimodal *k*-mer spectra, probably due to the focus of theoretical research on the distribution of individual *k*-mer counts. In hindsight, this observation is not so surprising since the spectrum is the ensemble of many different individual *k*-mer counts, each of which is an observation from a different, albeit related, distribution.

**Figure 3:**
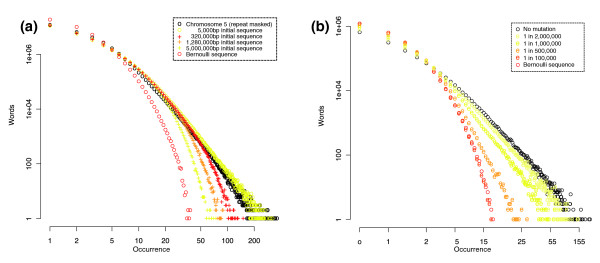
The copy/insert process does not always produce a heavy tailed spectrum. Effect of **(a) **increasing length of initial genome and **(b) **adding mutation to the copy/insert process. Graphs show the 11-mer spectrum of simulated genomes with (a) length equal to human chromosome 5, generated using a copy/insert process varying initial genome length, and (b) length 4 Mb, with a proportion of the bases mutated after each insert from an initial genome of 5,000 bp. As both axes are on a logarithmic scale, a distribution with a heavy 'power-law' tail (for example, no mutation) will tend to be a straight line, whereas lighter 'exponential' tails will bend downwards (for example, Bernoulli sequence). The sequences were constructed from an initial genome generated from a Bernoulli sequence with a CG content of 38.5%, matching human chromosome 5, by copying 33 base long chunks.

Low-order Markov models have some inherent limitations, especially when describing the 'heavy tail' of the genomic *k*-mer distribution. Simple Markov models are fit globally, and their parameters reflect average values across whole genomes, although they can be extended to include compositional heterogeneity by using a hidden Markov model framework [2]. Prominent examples of such heterogeneity are isochores, which can be millions of base-pairs long, and whose composition may differ substantially from the global composition. These often contain highly abundant *k*-mers that hardly exist elsewhere in the genome [19]. But, being relatively short (compared to the whole genome), these isochores hardly affect the model parameters; thus, these over-abundant *k*-mers will not be predicted by a Markov model. Csürös, Noé, and Kucherov [12] suggest that a copy/insert model can explain the heavy tail of *k*-mer spectra. Starting with a Bernoulli sequence, the copy/insert model generates a new sequence by inserting chunks of DNA, *m *bases long, from one position of a sequence into another. This copy and pasting is repeated many times until the final sequence is of the length required. For the simulations by Csürös *et al*. [12], chunks of 33 bases were copied in a genome of initial length 5,000 nucleotides (comparable in size to a small viral genome, like the bacteriophage phi-X). Figure 3a shows that the effect of increasing the size of chunk copied and inserted is that tails become more extreme, and that the effect of increasing the initial genome size is to lighten the tail. These results suggest that the apparent heavy tail produced by the copy/insert model may be an artifact of the short initial genome length used by Csürös *et al*. [12] for their simulations.

While the copy/insert process is deliberately designed to be abstract - a pedagogical tool to show how heavy tails can arise from biologically inspired processes - it neglects nucleotide mutation, which is a major cause of genomic change. To account for mutation, we randomly replaced a fixed percentage of nucleotides after each insertion. Figure 3b shows that the heavy tail of the copy/insert process collapses when even a small amount of mutation is present, and the resulting spectra are barely distinguishable from that of a Bernoulli sequence.

### CpG suppression and modality

Having established that Markov models can predict modalities, we can try and understand this phenomenon by looking at the di- and tri-nucleotide frequencies that are sufficient to define such models. The most outstanding property for the human genome is the low frequency of the CpG dimer, with P(G | C) = 0.048, and P(CpG) = 0.01. This well known phenomena, termed CpG suppression, occurs for all other mammals as well, but not for most other organisms.

**Figure 4:**
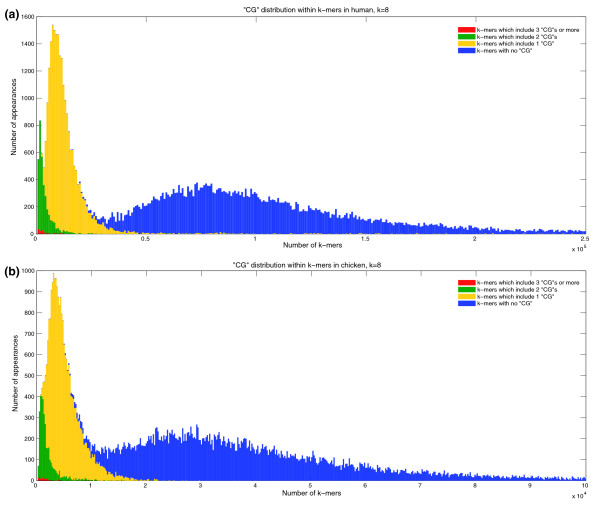
*k*-mer spectra of human and chicken, partitioned according to number of CpG dinucleotides in *k*-mers. *k*-mers with multiple CpGs are dominant among rare *k*-mers in the spectra of (a) human, and (b) chicken (*k *= 11). The 11-mer spectra are color-coded: blue, 11-mers with no CpG dinucleotides; yellow, exactly one CpG; green, exactly two CpG instances; red, three CpG instances or more.

If CpG is an uncommon dimer, then *k*-mers that contain it should be less frequent than those that do not. For human and chicken, those *k*-mers with higher CpG content appear further to the left of the histogram, as seen in Figure 4a, b. In these histograms, the *k*-mers having three or more instances of CpG are colored red, those with exactly two instances are colored green, those with exactly one instance are yellow, and those with no instances of CpG are blue. Additional data file 5 shows a similar aspect of the empirical word frequency spectra for four species that have multimodal and unimodal *k*-mer distributions: human, chicken, nematode and tetraodon (puffer fish). Words that include the dimer CpG are colored green, while all others are blue. For the multimodal distributions, the CpG-containing *k*-mers wholly occupy the left-most areas (rare words), but are almost absent from the regions representing more abundant *k*-mers. There is no such clear effect for the species with unimodal distributions.

These findings suggest the hypothesis that CpG suppression is what determines modality. It turns out this is incorrect, and CpG suppression by itself does not fully account for multimodal word frequencies. This is because there are several unicellular species that exhibit CpG suppression, yet have unimodal word frequencies. One such organism is the protozoa *Tetrahymena thermophila*, which has P(CpG) = 0.005 yet has a unimodal distribution. We note that the difference in modalities is not due to the difference in genome lengths. The genome of *T. thermophila *is 97 Mb long, comparable to the 108 Mb of human chromosome 12, yet all human chromosomes have multimodal spectra.

To further analyze the relations between CpG suppression and modality, we looked at the values of both GC-content and a measure of suppression. The GC-content of a string of DNA is simply the fraction of the letters that are C plus those that are G. As a measure of suppression, we use *ρ *as defined by Karlin *et al*. [20] as the ratio of the empirical frequency of a dimer to the product of the frequencies of its constituent monomers (the expected frequency if they combined independently to form dimers). For the CpG dimer, the relevant ratio is:

If the occurrences of C and G were independent, *ρ*_CG _would be 1; genomes exhibiting suppression will have *ρ*_CG _much less than 1. For human, *ρ*_CG _= 0.24, for opossum it is 0.13, for lizard 0.3 and for frog 0.34. Low values are also attained for some protozoa (for example, *Entamoeba histolytica*, 0.30) and archea (for example, *Methanococcus jannaschii*, 0.32; *Methanosphaera stadtmanae*, 0.27). Values of *ρ*_CG _exceeding 1 are infrequent but do exist; for example, 1.15 for the red flour beetle *Tribolium castaneum*, and 1.64 for the honey bee *Apis mellifera*.

**Figure 5:**
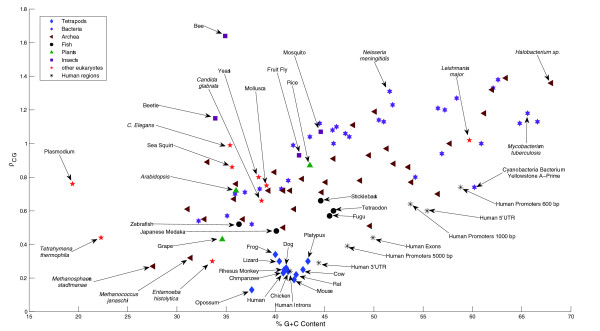
Distribution of CpG suppression and CG content of genomes studied. Distribution of CpG suppression, measured by *ρ*_CG_, against the CG content of many genomes; evolutionarily interesting groups are differentiated by symbols. Notice that the tetrapods, all of whose genomes have multimodal *k*-mer spectra, form a tight grouping in the lower middle part of the graph. The three closest partitions of human genomic sequence, the introns, 3' UTRs and promoter regions (5,000 bp upstream of the 5' UTR), also have multimodal *k*-mer spectra. Other nearby genomes, *Entamoeba histolytica *and Japanese medaka (*Oryzias latipes*), as well as other kinds of human genomic sequences (exons, 5' UTRs, and shorter promotor regions) have unimodal spectra.

Figure 5 plots the percentage GC-content against *ρ*_CG _for a set of representative species (for lack of space, not all studied bacteria and archea are included). The species that exhibit multimodal *k*-mer histograms cluster closely in this plot. We can also see that some species, such as the archeon *Methanosphaera stadtmanae *and *Methanococcus jannaschi *and the protozoan *Entamoeba hystolytica*, all have *ρ*_CG _< 0.33, values that are similar to lizard and smaller than frog. (*T. thermophila *mentioned above has *ρ*_CG _= 0.44 despite having P(CpG) = 0.005, due to its low GC-content of 22.3%.) Yet, due to lower GC-content, they are not part of the multimodal cluster of species. This indicates that what determines multimodality is a combination of GC-content in the range 35% to 45%, and *ρ*_CG _values smaller than 0.4.

## Conclusions

It is of interest to examine the modalities of the word frequency distributions from an evolutionary perspective. All archeal and bacterial species exhibit unimodal *k*-mer distributions whereas there is a difference amongst the eukaryota. All mammals, including human, chimp, mouse, dog, cow, opossum (a non-placental mammal), and platypus (a monotreme, egg-laying mammal) exhibit multimodal *k*-mer distributions. Non-mammals that exhibit multimodal distributions are chicken, lizard, and frog. All these 'multimodal' species are tetrapods. The next sister clade in the tree of life contains the bony fish but none of the five sequenced bony fish (zebrafish, fugu, tetraodon, stickleback, and Japanese medaka) have multimodal *k*-mer spectra, in common with all other eukaryota except tetrapods. Following this observation, we predict that the genomes of additional tetrapods currently being sequenced, such as the alligator (*Alligator mississippiensis*), will also be multimodal.

We find the fact that low order Markov models are capable of producing multimodal spectra, and, furthermore, that their modalities match those of the actual genomes, to be rather unexpected. We note that the modalities of the Markov model distributions were essentially found experimentally (by simulation). It may be possible to find the modalities, and other global properties of the *k*-mer spectra, analytically (from just the model's parameters).

Which distribution better fits the genome sequences - low-order Markov models, or a DPLN distribution? The DPLN distribution is the ratio of two Pareto distributions times a log-normal [13] and has a single mode. Consequently, it cannot describe multimodal distributions such as the tri-modal distribution observed for human 11-mers (Figure 1). Csürös *et al*. use a mixture of four separate DPLN distributions, separating the *k*-mers into four groups by CpG content (0, 1, 2 and 3+ occurrences, respectively). Such mixtures can indeed fit a tri-modal distribution, but this raises the question of whether a simple generative model cannot capture these phenomena as well.

We remark that our own simulation of the 'copy/insert' process of Csürös *et al*. does not necessarily produce a heavy tailed *k*-mer spectrum and, in fact, the resulting *k*-mer spectrum can have a light 'exponential' tail similar to a Bernoulli sequence. The reason for the difference may lie in the length and *k*-mer content of the initial sequence used to prime the copy/insert process; too short a sequence and the few *k*-mers it does contain become abundant before the new *k*-mers can be generated by splicing. The lack of robustness to mutation of the heavy-tail produced by the copy/insert process suggests that this process does not explain how such tails arise in nature.

Lastly, we note that while the DPLN fits the spectrum well, noticeably so at the tails of the distribution, it is not a generative model: the shape of the distribution fits but it says little about the probability of observing a particular *k*-mer. Generative models like the Markov models predict which *k*-mers are likely to be rare or abundant, whereas the DPLN makes no such prediction.

## Materials and methods

Genomic sequences were obtained from the standard sequence archives for each organism, listed in Additional data file 7, and masked for repeats. In several cases, the effective sequence length we report differs substantially from the accepted length - platypus being a noticeable outlier - due to the genome being incompletely sequenced or having large sections masked as highly repetitive. Nucleotides that are ambiguous or masked were considered to be breaks in the sequence and so any *k*-mer containing them was discarded and not enumerated. The *k*-mers for each genome where enumerated using simple programs written by the authors, from which the spectra and associated statistics are calculated.

When we use the same *k *for shorter genome length ℓ, we get a larger proportion of words at the low end of the curve. To account for this, smaller values of *k *are used for shorter genomes. Since the total number of possible *k*-mers is 4^*k*^, if we take *k *= log_4 _ℓ then the mean number of occurrences in an ℓ long genome is 1. This indicates that, to normalize for the differing lengths of each genome, *k *should be chosen proportional to log_4 _ℓ. Specifically, we looked at values of *k *= ⌈0.7 log_4_ℓ⌉ (the smallest integer greater than 0.7 log_4 _ℓ). Simple Markov models of the type described can be fitted through counts of short *k*-mers. The transition frequencies, the proportion of times we see each nucleotide in the genome given the previous *k *nucleotides in the genome, are the maximum likelihood estimates for the transition probabilities of a *k*-order Markov model [21]. To fit a zero-order model, only the nucleotide frequencies of each genome are needed; dinucleotide frequencies alone are needed for first-order models, trinucleotide frequencies for second-order, and so on. These frequencies were calculated using the programs written to determine the *k*-mer spectra of each genome. Again, bases that are ambiguous or masked were considered as breaks in the sequence and so not enumerated. Sequences were then simulated from these models using standard techniques. The copy/insert process was simulated using the authors' own programs. Following Csürös *et al*. [12], a (composition biased) Bernoulli sequence was used to generate an initial genome. Chunks of genome 33 bp long were chosen uniformly at random, copied, and inserted into a random place in the genome; this step was repeated until the genome reached a specified length. Unless otherwise stated, the initial genome was 5,000 bp and the final length was about 67 Mb (comparable to repeat masked human chromosome 5) to maintain consistency with [12]. To investigate the effect of mutation, we adapted the copy/insert process so that every insertion was followed by mutation of a fixed proportion of the nucleotides, chosen uniformly at random and replaced with random nucleotides from a (composition biased) Bernoulli distribution.

## Abbreviations

DPLN: double Pareto log-normal; UTR: untranslated region.

## Authors' contributions

All authors contributed to the design of this study. The initial exploration of *k*-mer modality was done by TM, along with the investigation of the copy/insert model. YL investigated the modalities of all genomes listed in Additional data file 7, supervised by BC and DH, and the taxonomic distribution of multimodality. BC and TM prepared the paper.

## Additional data files

The following additional data are available with the online version of this paper:

Spectra of individual human chromosomes (Additional data file 1); whole genome spectra of human and opossum (Additional data file 2); whole genome spectra of nine non-tetrapodal species (Additional data file 3); spectra of additional, non-mammalian tetrapod whole genomes (Additional data file 4); the distribution of 8-mers containing the dimer CpG in four eukaryotic species (Additional data file 5); spectra of specific human genomic regions (Additional data file 6); organisms whose genomes were used in this study, along with additional information about each (Additional data file 7).

## Supplementary Material

Additional data file 1 On the top are 9-mer spectra of human chromosomes (left to right) 1, 6, 20. At the bottom are 11-mer spectra of human chromosomes (left to right) 1, 6, 20. All six spectra are multimodal. Click here for file

Additional data file 2 Whole genome spectra of human (top) and opossum (bottom); 9-mers (left) and 11-mers (right). All four spectra are multimodal. Click here for file

Additional data file 3 From top-left: *Escherichia coli*, *Aeropyrum pernix*, zebrafish (*Danio rerio*), pufferfish (*Tetraodon nigroviridis*), *Arabidopsis thaliana*, bee (*Apis mellifera*), nematode (*Caenorhabditis elegans*), yeast (*Saccharomyces cerevisiae*), and sea squirt (*Ciona savignyi*). All spectra are unimodal. Click here for file

Additional data file 4 Chicken (*k *= 10), platypus (*Ornithorhynchus anatinus*, an egg laying mammal; *k *= 10), frog (*k *= 11), and lizard (*k *= 11). All four *k*-mer spectra are multimodal. Click here for file

Additional data file 5 Each plot is partitioned according to 8-mers that contain the CpG dimer (colored green), and those that do not (colored blue). The green ones comprise the left-most part in the multimodal spectra for (a) human, and (b) chicken. In the two other, non-tetrapodal species, (c) *C. elegans *(nematode), and (d) pufferfish, there is no such effect. Click here for file

Additional data file 6 Whole genome (*k *= 10), all introns (*k *= 10), all 3' UTRs (*k *= 10), all exons (*k *= 9), all 5' UTRs (*k *= 8), all 600 base long promotors (*k *= 6), all 1,000 base long promotors (*k *= 7), all 5,000 base long promotors (*k *= 7). Click here for file

Additional data file 7 Additional information includes taxonomical classification, usable length, percentage C+G content, CpG suppression (measured by *ρ*_CG_) and whether the *k*-mer spectrum is observed to have unimodal or multimodal behavior. It also specifies web sites from which sequences were downloaded [22-27], and where different functional genomic regions were downloaded from [28-30]. Click here for file
