# Supplementary material for: Genomic DNA k-mer spectra: models and modalities
Source: Genome Biol. 2009 Oct 8;10(10):R108. doi: 10.1186/gb-2009-10-10-r108 (PMC2784323; doi:10.1186/gb-2009-10-10-r108)
Supplement: Additional data file 1 — On the top are 9-mer spectra of human chromosomes (left to right) 1, 6, 20. At the bottom are 11-mer spectra of human chromosomes (left to right) 1, 6, 20. All six spectra are multimodal. [file gb-2009-10-10-r108-S1.pdf]

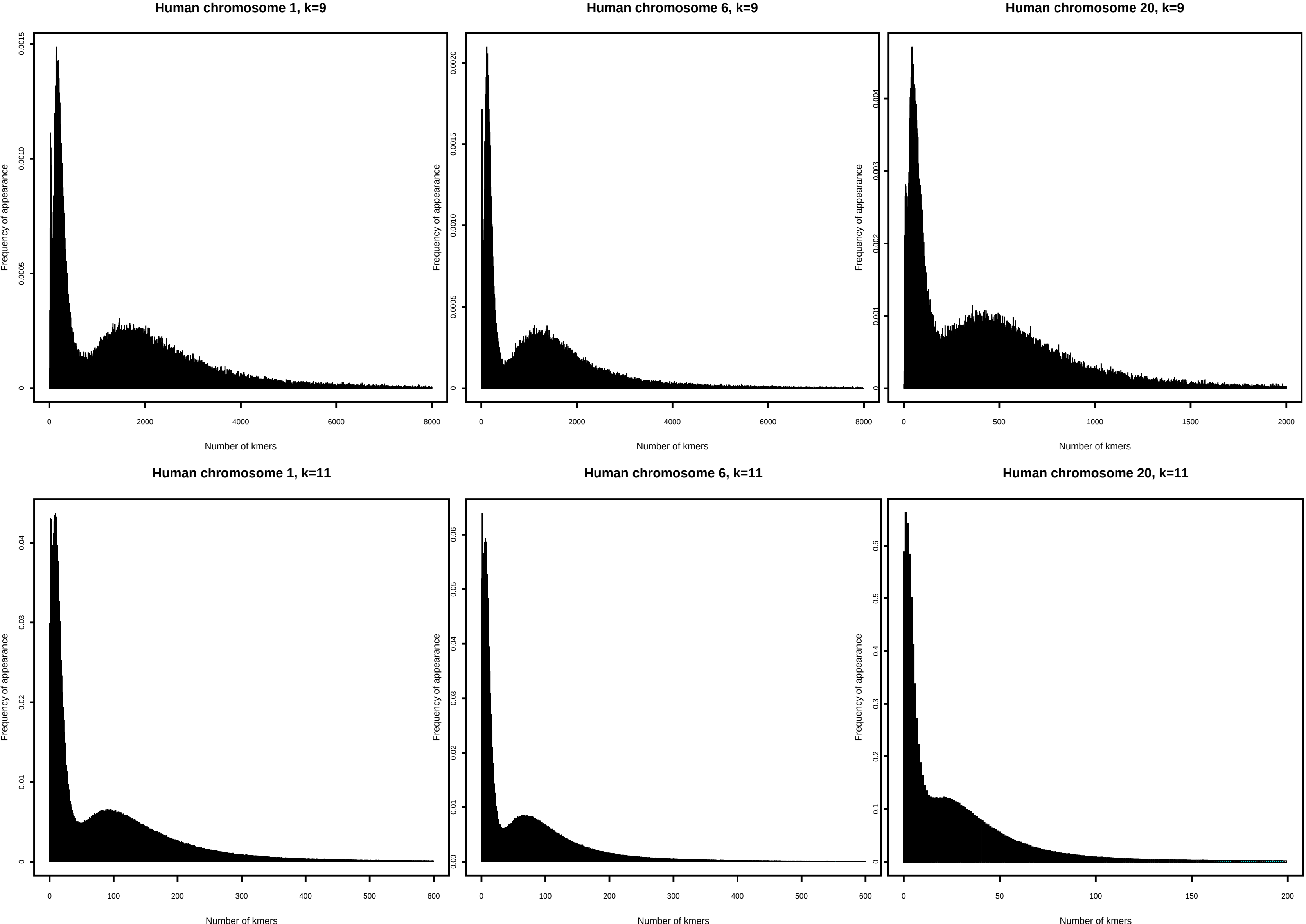

Human chromosome 1, k=9
Human chromosome 6, k=9
Human chromosome 20, k=9
Human chromosome 1, k=11
Human chromosome 6, k=11
Human chromosome 20, k=11
Frequency of appearance
Number of kmers
0
0.0005
0.0010
0.0015
0.0020
0.001
0.002
0.003
0.004
0.00
0.01
0.02
0.03
0.04
0.05
0.06
0.1
0.2
0.3
0.4
0.5
0.6
2000
4000
6000
8000
500
1000
1500
100
200
300
400
600
50
150
