# Supplementary figures and images for: Genomic DNA k-mer spectra: models and modalities
Source: Genome Biol. 2009 Oct 8;10(10):R108. doi: 10.1186/gb-2009-10-10-r108 (PMC2784323; doi:10.1186/gb-2009-10-10-r108)

Human, k=9

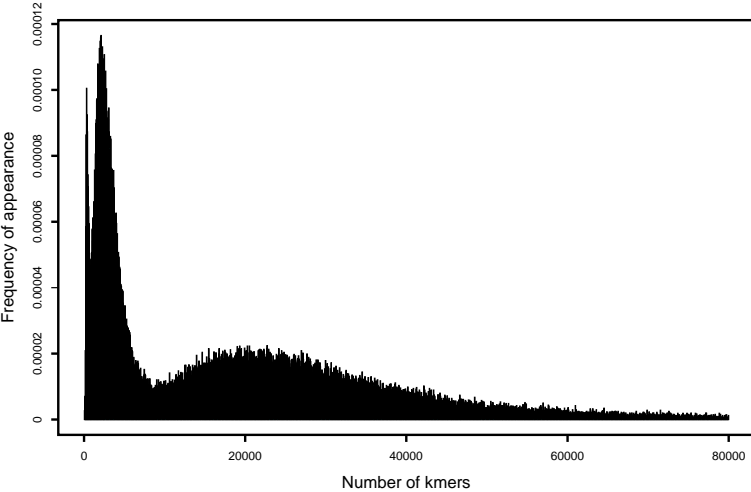

Human, k=11

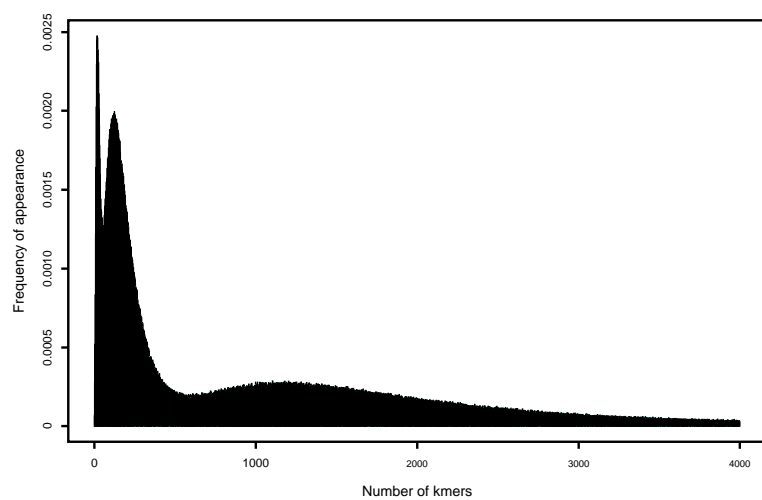

Opossum, k=9

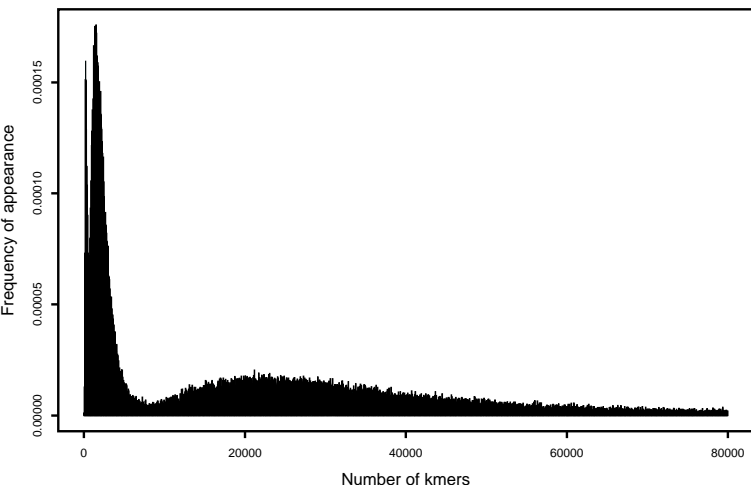

Opossum, k=11

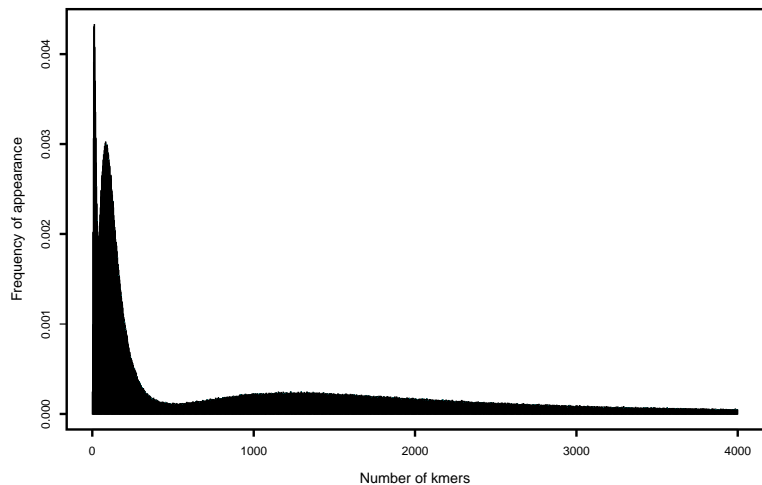

Supplement: Additional data file 2 — Whole genome spectra of human (top) and opossum (bottom); 9-mers (left) and 11-mers (right). All four spectra are multimodal. [file gb-2009-10-10-r108-S2.pdf]

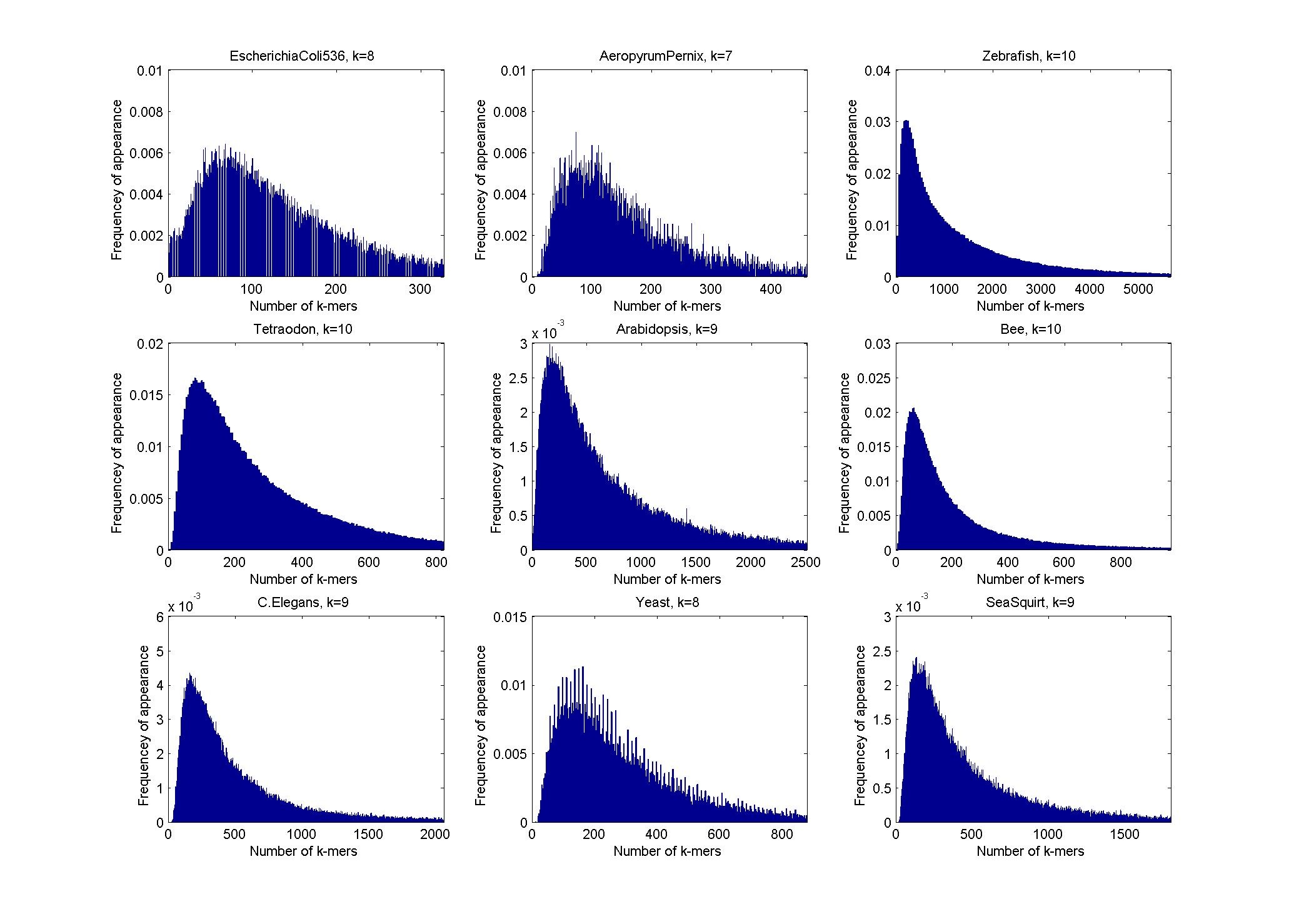

Supplement: Additional data file 3 — From top-left: Escherichia coli, Aeropyrum pernix, zebrafish (Danio rerio), pufferfish (Tetraodon nigroviridis), Arabidopsis thaliana, bee (Apis mellifera), nematode (Caenorhabditis elegans), yeast (Saccharomyces cerevisiae), and sea squirt (Ciona savignyi). All spectra are unimodal. [file gb-2009-10-10-r108-S3.jpeg]

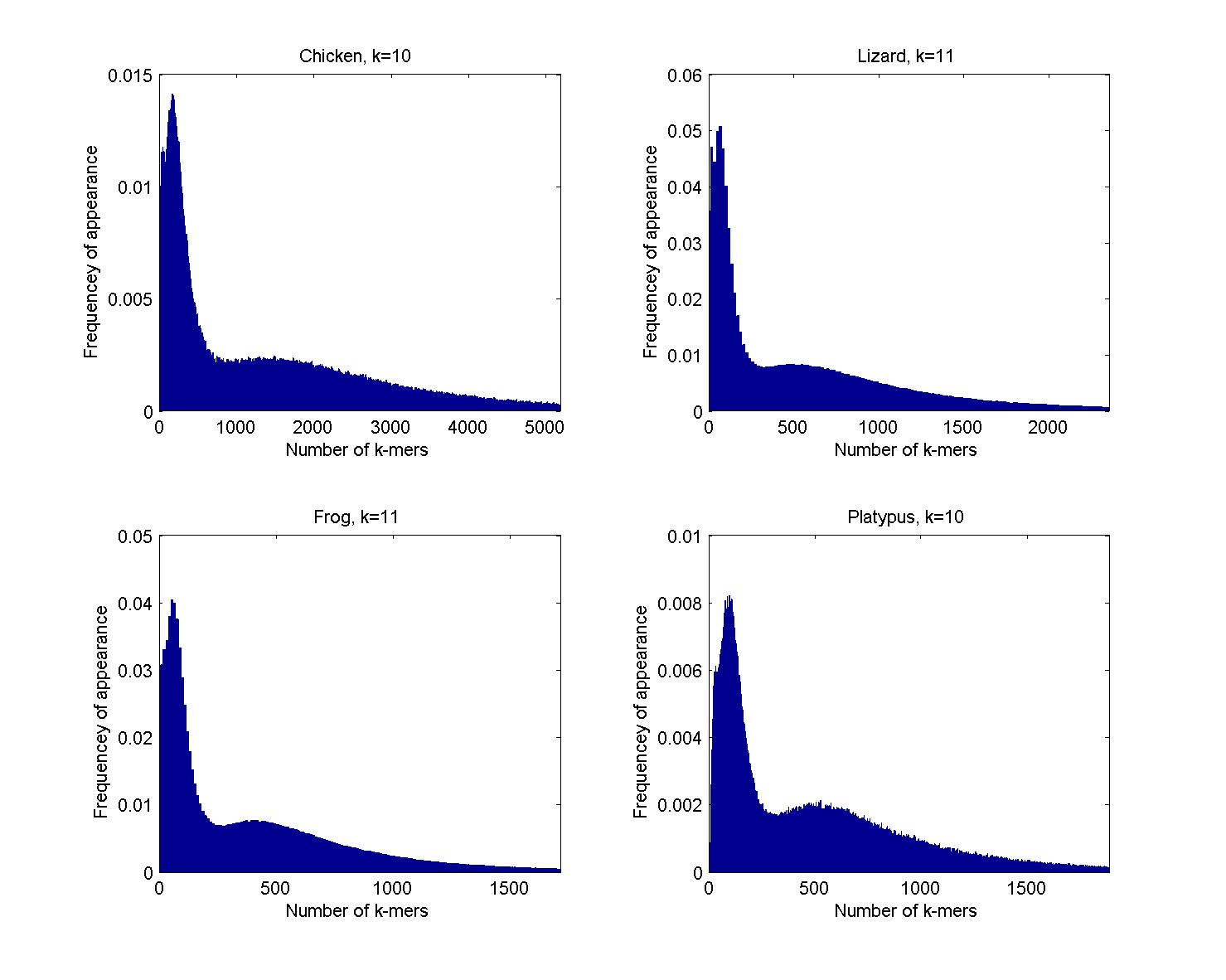

Supplement: Additional data file 4 — Chicken (k = 10), platypus (Ornithorhynchus anatinus, an egg laying mammal; k = 10), frog (k = 11), and lizard (k = 11). All four k-mer spectra are multimodal. [file gb-2009-10-10-r108-S4.jpeg]

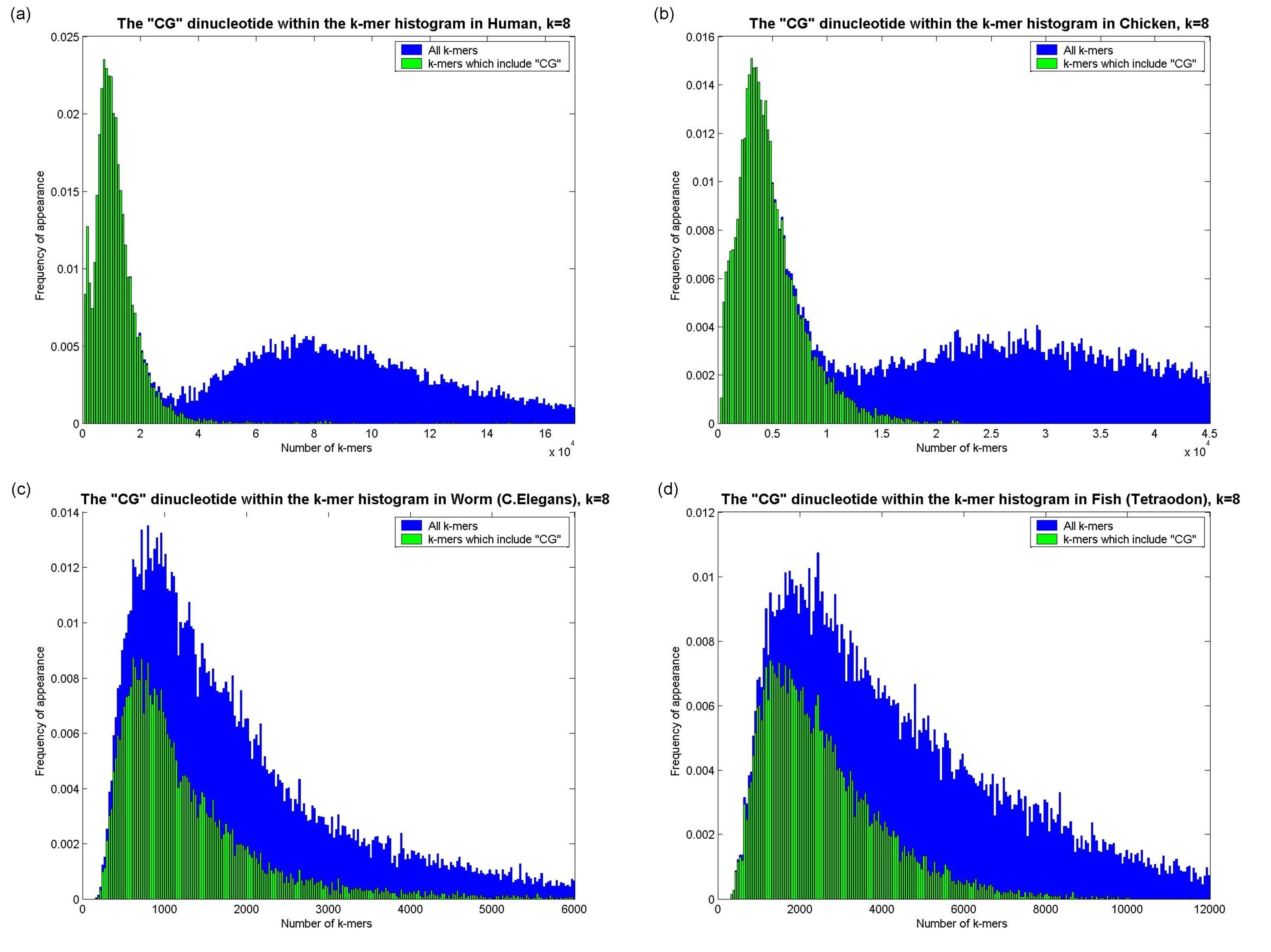

Supplement: Additional data file 5 — Each plot is partitioned according to 8-mers that contain the CpG dimer (colored green), and those that do not (colored blue). The green ones comprise the left-most part in the multimodal spectra for (a) human, and (b) chicken. In the two other, non-tetrapodal species, (c) C. elegans (nematode), and (d) pufferfish, there is no such effect. [file gb-2009-10-10-r108-S5.jpeg]

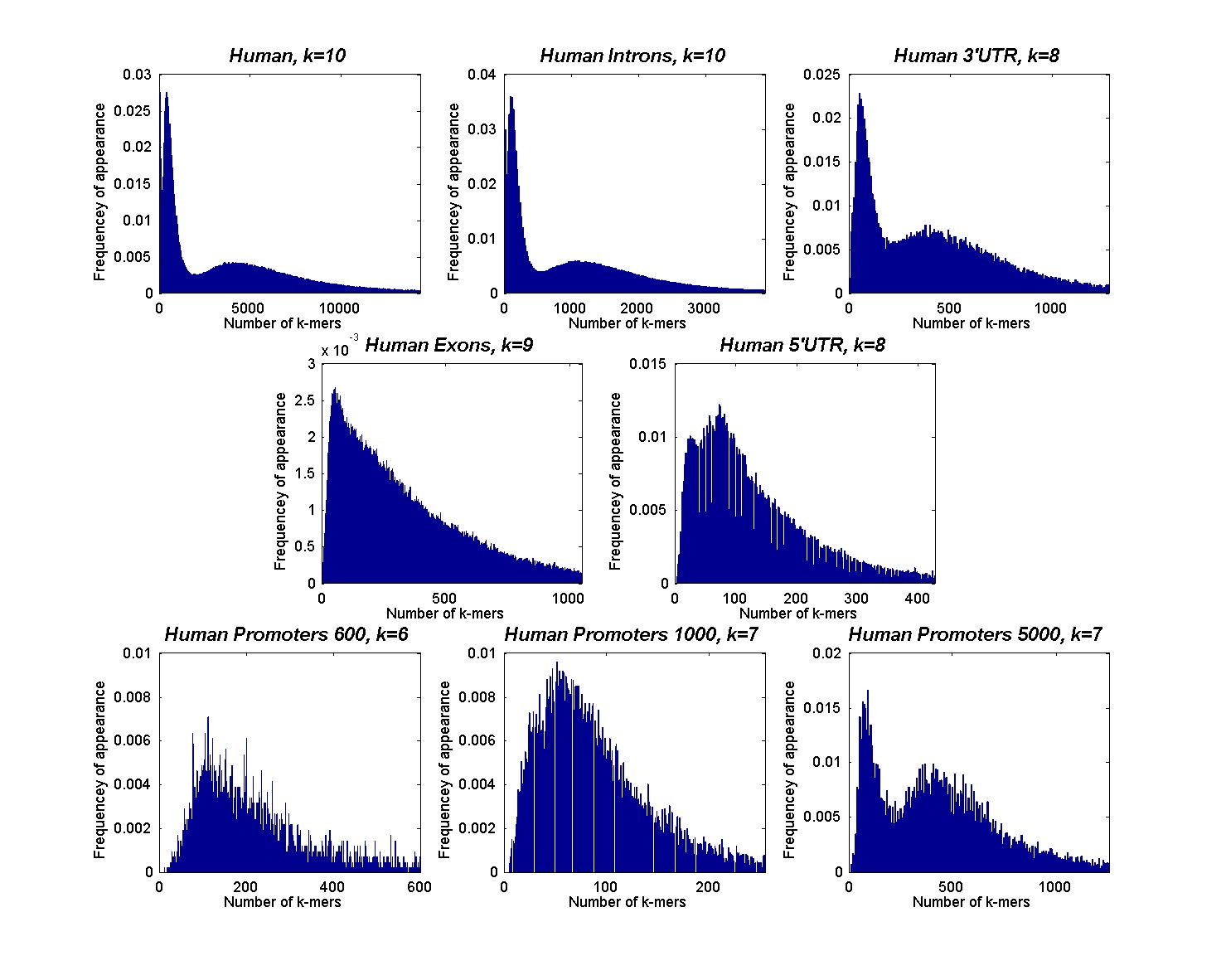

Supplement: Additional data file 6 — Whole genome (k = 10), all introns (k = 10), all 3' UTRs (k = 10), all exons (k = 9), all 5' UTRs (k = 8), all 600 base long promotors (k = 6), all 1,000 base long promotors (k = 7), all 5,000 base long promotors (k = 7). [file gb-2009-10-10-r108-S6.jpeg]
