# Supplementary material for: Genomic DNA k-mer spectra: models and modalities
Source: Genome Biol. 2009 Oct 8;10(10):R108. doi: 10.1186/gb-2009-10-10-r108 (PMC2784323; doi:10.1186/gb-2009-10-10-r108)
Supplement: Additional data file 7 — Additional information includes taxonomical classification, usable length, percentage C+G content, CpG suppression (measured by ρCG) and whether the k-mer spectrum is observed to have unimodal or multimodal behavior. It also specifies web sites from which sequences were downloaded [22-27], and where different functional genomic regions were downloaded from [28-30]. [file gb-2009-10-10-r108-S7.pdf]

Table 1 - Genomes and Web Sites Used

The following tables list the organisms whose genomes were used in this study, along with additional information about each of them: taxonomical classification, usable length, percentage C+G content, CpG suppression (measured by $\rho_{\texttt{CG}}$) and whether the k-mer spectrum is observed to have uni-modal or multi-modal behaviour.

Genomic data of the different species was downloaded from the following sites:

- **Ensemble ftp site** [22]
- **NCBI ftp site** [23]
- **TIGR ftp site** [24]
- **UCSC website** [25]
- **HGSC ftp site** [26]
- **Genoscope website** [27]

Data corresponding to various human functional genomic regions was downloaded from:

- **Human exons and introns** [28]
- **Human 3'UTRs and 5'UTRs** [29]
- **Human gene promotors** [30]

For some species referred to in this work, fully assembled genomes were not available. In such cases we used the available material (contigs, scaffolds, etc), making the assumption that the final assemblies will not differ significantly from these preliminary files in terms of the k-mer statistics.

**Species (Archea)**	**Classification**	**Length**	**%G+C**	ρ_{CG}	**dist. type**
AeropyrumPernix	Archea	1,509,911	56.5	0.70	unimodal
ArchaeoglobusFulgidus	Archea	2,076,061	48.6	0.78	unimodal
HaloarculaMarismortui	Archea	3,208,489	62.0	1.32	unimodal
HalobacteriumSp	Archea	1,887,389	68.0	1.36	unimodal
HaloquadratumWalsbyi	Archea	2,925,353	47.9	1.11	unimodal
HyperthermusButylicus	Archea	1,509,911	53.8	0.77	unimodal
MethanobacteriumThermoautotrophicum	Archea	1,604,247	49.6	0.51	unimodal
MethanococcoidesBurtonii	Archea	2,453,539	40.8	0.72	unimodal
MethanococcusJannaschii	Archea	1,604,238	31.4	0.32	unimodal
MethanococcusMaripaludis	Archea	1,509,911	33.1	0.89	unimodal
MethanocorpusculumLabreanum	Archea	1,698,650	50.1	1.19	unimodal
MethanopyrusKandleri	Archea	1,604,247	61.2	1.18	unimodal
MethanosaetaThermophila	Archea	1,792,986	53.5	0.86	unimodal
MethanosarcinaAcetivorans	Archea	5,473,296	42.7	0.79	unimodal
MethanosarcinaBarkeri	Archea	4,624,004	39.3	0.72	unimodal
MethanosarcinaMazei	Archea	3,869,048	41.5	0.72	unimodal
MethanosphaeraStadtmanae	Archea	1,604,247	27.6	0.27	unimodal
MethanospirillumHungatei	Archea	3,302,831	45.1	0.77	unimodal
NanoarchaeumEquitans	Archea	377,477	31.1	0.61	unimodal
NatronomonasPharaonis	Archea	2,453,539	63.4	1.39	unimodal
PicrophilusTorridus	Archea	1,415,508	36.0	0.76	unimodal
PyrobaculumAerophilum	Archea	2,076,061	51.3	0.97	unimodal
PyrobaculumIslandicum	Archea	1,698,650	49.5	0.93	unimodal
PyrococcusAbyssi	Archea	1,604,247	44.7	0.71	unimodal
PyrococcusFuriosus	Archea	1,792,986	40.8	0.50	unimodal
PyrococcusHorikoshii	Archea	1,604,247	41.9	0.61	unimodal
SulfolobusAcidocaldarius	Archea	2,076,061	36.7	0.55	unimodal
SulfolobusSolfataricus	Archea	2,831,017	35.8	0.67	unimodal
SulfolobusTokodaii	Archea	2,547,942	32.8	0.55	unimodal
ThermococcusKodakaraensis	Archea	1,981,725	52.0	0.88	unimodal
ThermofilumPendens	Archea	1,698,650	57.7	1.00	unimodal
ThermoplasmaAcidophilum	Archea	1,415,508	45.9	0.91	unimodal
ThermoplasmaVolcanium	Archea	1,509,911	39.9	0.83	unimodal

**Species (Bacteria)**	**Classification**	**Length**	**%G+C**	ρ_{CG}	**dist. type**
AcidobacteriaBacteriumEllin345	Bacteria	5,378,893	58.4	1.27	unimodal
BacillusSubtilis	Bacteria	3,963,384	43.5	1.04	unimodal
BrucellaMelitensis	Bacteria	3,114,055	57.2	1.20	unimodal
BurkholderiaXenovoransLB400	Bacteria	9,247,928	62.6	1.38	unimodal
ChlamydophilaPneumoniaeAR39	Bacteria	1,132,366	40.6	0.73	unimodal
ChlorobiumTepidumTLS	Bacteria	1,981,718	56.5	1.21	unimodal
ChromobacteriumViolaceum	Bacteria	4,529,601	64.8	1.12	unimodal
CyanobacteriaBacteriumYellowstoneA-Prime	Bacteria	2,736,681	60.2	0.74	unimodal
EscherichiaColi536	Bacteria	4,718,340	50.5	1.14	unimodal
FrancisellaTularensisHolarctica	Bacteria	1,792,986	32.2	0.54	unimodal
GeobacterSulfurreducens	Bacteria	3,585,973	60.9	1.00	unimodal
HelicobacterHepaticus	Bacteria	1,698,650	35.9	0.70	unimodal
IdiomarinaLoihiensisL2TR	Bacteria	2,642,278	47.1	1.06	unimodal
LactobacillusPlantarum	Bacteria	3,114,092	44.5	1.12	unimodal
LegionellaPneumophilaLens	Bacteria	3,208,092	38.4	0.73	unimodal
MagnetococcusMC-1	Bacteria	4,435,265	54.2	0.80	unimodal
MarinobacterAquaeoleiVT8	Bacteria	4,529,611	56.9	0.94	unimodal
MycobacteriumTuberculosis	Bacteria	4,151,874	65.6	1.18	unimodal
NeisseriaMeningitidisFAM18	Bacteria	2,076,061	51.6	1.31	unimodal
NitrobacterWinogradskyiNb-255	Bacteria	3,208,495	62.1	1.33	unimodal
NostocSp	Bacteria	6,888,767	41.3	0.78	unimodal
PhotobacteriumProfundumSS9	Bacteria	6,039,402	41.8	0.99	unimodal
ProchlorococcusMarinusNATL2A	Bacteria	1,698,650	35.1	0.57	unimodal
RalstoniaEutrophaH16	Bacteria	6,605,672	66.6	1.13	unimodal
SaccharophagusDegradans2-40	Bacteria	4,812,743	45.8	1.08	unimodal
SalmonellaTyphi	Bacteria	4,907,083	51.9	1.23	unimodal
ShewanellaOneidensis	Bacteria	4,907,081	45.9	1.00	unimodal
ShigellaDysenteriae	Bacteria	4,340,901	51.0	1.13	unimodal
StreptococcusMutans	Bacteria	1,887,389	36.9	0.71	unimodal
ThermoanaerobacterTengcongensis	Bacteria	2,547,942	37.6	0.52	unimodal
VibrioCholerae	Bacteria	3,774,608	47.5	1.04	unimodal
ZymomonasMobilisZM4	Bacteria	1,887,389	46.2	1.10	unimodal
PlasmodiumFalciparum (malaria)	Bacteria (Protozoa)	21,798,142	19.4	0.76	unimodal
TetrahymenaThermophila	Bacteria (Protozoa)	98,669,430	22.3	0.44	unimodal
LeishmaniaMajor	Bacteria (Protozoa)	31,116,157	59.7	1.02	unimodal
EntamoebaHistolytica	Bacteria (Protozoa)	12,298,665	33.6	0.30	unimodal

**Species (Eukaryotes)**	**Classification**	**Length**	**%G+C**	ρ_{CG}	**dist. type**
CaenorhabditisElegans (worm)	Nematode	95,970,454	35.4	0.99	unimodal
SaccharomycesCerevisiae (yeast)	Fungi	11,512,682	38.3	0.80	unimodal
CandidaGlabrata (haploid yeast)	Fungi	11,700,869	38.6	0.66	unimodal
BiomphalariaGlabrata (mollusca)	Mollusk	48,845,378	39.1	0.75	unimodal
CionaIntestinalis (sea squirt)	Tunicate	83,180,064	35.6	0.86	unimodal
ArabidopsisThaliana (arabidopsis)	Plant	113,813,113	36.0	0.72	unimodal
OryzaSativa (rice)	Plant	385,822,552	43.5	0.87	unimodal
VitisVinifera (grape)	Plant	468,739,222	34.6	0.43	unimodal
DrosophilaMelanogaster (fly)	Insect	115,134,437	42.4	0.93	unimodal
AnophelesGambiae (mosquito)	Insect	213,534,127	44.6	1.07	unimodal
ApisMellifera (bee)	Insect	175,355,887	34.9	1.64	unimodal
TriboliumCastaneum (beetle)	Insect	145,486,340	33.9	1.15	unimodal
DanioRerio (zebrafish)	Bony Fish	996,230,784	36.3	0.52	unimodal
TakifuguRubripes (fugu)	Bony Fish	329,961,080	45.5	0.57	unimodal
TetraodonNigroviridis (pufferfish)	Bony Fish	177,300,831	45.9	0.60	unimodal
GasterosteusAculeatus (stickleback)	Bony Fish	424,233,346	44.6	0.66	unimodal
OryziasLatipes (Japanese Medaka)	Bony Fish	552,716,066	40.1	0.48	unimodal
AnolisCarolinensis (lizard)	Reptile	1,676,035,836	40.4	0.30	multi-modal
XenopusTropicalis (frog)	Amphibian	1,288,438,558	40.0	0.34	multi-modal
GallusGallus (chicken)	Bird	942,474,046	41.3	0.24	multi-modal
OrnithorhynchusAnatinus (platypus)	Mammal	388,840,627	43.3	0.30	multi-modal
BosTaurus (cow)	Mammal	1,408,036,795	42.8	0.25	multi-modal
CanisFamiliaris (dog)	Mammal	2,187,364,344	41.1	0.26	multi-modal
RattusNorvegicus (rat)	Mammal	2,327,934,313	42.1	0.22	multi-modal
MacacaMulatta (rhesus monkey)	Mammal	2,503,126,668	40.9	0.25	multi-modal
MonodelphisDomestica (opossum)	Mammal	3,287,333,976	37.6	0.13	multi-modal
MusMusculus (mouse)	Mammal	2,387,461,979	41.9	0.19	multi-modal
PanTroglodytes (chimpanzee)	Mammal	2,596,334,645	40.8	0.23	multi-modal
HomoSapiens (human)	Mammal	2,735,501,651	40.9	0.24	multi-modal
HumanIntrons (human regions)		1,368,981,774	41.5	0.24	multi-modal
Human3'UTR (human regions)		30,577,457	44.4	0.29	multi-modal
HumanExons (human regions)		108,962,293	49.9	0.44	unimodal
Human5'UTR (human regions)		10,160,999	55.4	0.60	unimodal
HumanPromoters600 (human regions)		1,025,512	58.8	0.74	unimodal
HumanPromoters1000 (human regions)		1,762,896	53.7	0.64	unimodal
HumanPromoters5000 (human regions)		8,753,016	47.3	0.39	multi-modal
